# Albuminuria, serum antioxidant enzyme levels and markers of hemolysis and inflammation in steady state children with sickle cell anemia

**DOI:** 10.1186/s12882-016-0398-0

**Published:** 2016-11-17

**Authors:** Karen E. Itokua, Jean Robert Makulo, François B. Lepira, Michel N. Aloni, Pépé M. Ekulu, Ernest K. Sumaili, Justine B. Bukabau, Vieux M. Mokoli, Augustin L. Longo, François M. Kajingulu, Chantal V. Zinga, Yannick M. Nlandu, Yannick M. Engole, Pierre Z. Akilimali, René M. Ngiyulu, Jean Lambert Gini, Nazaire M. Nseka

**Affiliations:** 1 Division of Hematology, Oncology and Nephrology, Department of Pediatric, University of Kinshasa Hospital, University of Kinshasa, Kinshasa, Democratic Republic of the Congo; 2 Nephrology Unit, Department of Internal Medicine, University of Kinshasa Hospital, University of Kinshasa, Kinshasa, Democratic Republic of the Congo; 3 Kinshasa School of Public Health, University of Kinshasa, Kinshasa, Democratic Republic of the Congo; 4 Faculty of Medicine, University of Kinshasa Hospital, Kinshasa, Democratic Republic of the Congo

**Keywords:** Sickle cell anemia, Albuminuria, GPx, Cu-Zn SOD, Lactate dehydrogenase, Inflammation

## Abstract

**Background:**

Oxidative stress is thought to be involved in the pathogenesis of microalbuminuria in Sickle cell anemia (SCA). Antioxidant enzymes such as glutathione peroxidase (GPx) and Cu-Zn superoxide dismutase (SOD) may play an important protective role. This study aimed to evaluate the association between albuminuria and these two antioxidant enzymes.

**Methods:**

We consecutively recruited Steady state children aged between 2 and 18 years old with established diagnosis of homozygous SCA in two hospitals of Kinshasa/DR Congo. The relationship between Urinary Albumin Creatinine Ratio (UACR) and other variables of interest (age, systolic blood pressure, diastolic blood pressure, plasma GPx and Cu-Zn SOD, free plasmatic hemoglobin, LDH, indirect bilirubin, white blood cells (WBC), percentage of fetal hemoglobin, serum iron, ferritin, CRP) was analyzed by Bivariate correlation (Pearson’s correlation coefficient). Microalbuminuria was defined by urine albumin/creatinine ratio between 30 and 299 mg/g.

**Results:**

Seventy Steady state Black African children with SCA (56% boys; average age 9.9 ± 4.3 years; 53% receiving hydroxyurea) were selected. Prevalence of microalbuminuria was 11.8%. LDH (*r* = 0.260; *p* = 0.033) and WBC count (*r* = 0.264; *p* = 0.033) were positively correlated with UACR whereas GPx (− 0.328; *p* = 0.007) and Cu-Zn SOD (− 0.210; *p* = 0.091) were negatively correlated with UACR.

**Conclusions:**

Albuminuria is associated with decreased antioxidant capacity and increased levels of markers of hemolysis and inflammation. Therefore, strategies targeting the reduction of sickling and subsequent hemolysis, oxidative stress and inflammation could help preventing or at least delaying the progression of kidney disease in SCA children.

## Background

Sickle cell anemia (SCA) is a hemoglobinopathy characterized by hemolytic anemia, increased susceptibility to infections and vaso-occlusion leading to a reduced patient’s quality of life and life expectancy [1]. Oxidative stress has been reported to play a significant role in the pathophysiology of hemolysis, vaso-occlusion and subsequent organ damage including the kidneys [1–3]. Sickling and subsequent hemolysis and anemia increase superoxide anion and other reactive oxygen species (ROS) production and impair ROS scavenging enzymes levels and activity in SCA patients [1]. Low ROS scavenging enzymes activity, due to either low intake of vitamins or impaired synthesis of enzymes because of cofactors deficiency [4], is thought to play the main role in the pathogenesis of oxidative stress-induced vascular complications and microalbuminuria [5]. Indeed, lower activities of superoxide dismutase (SOD), glutathione peroxidase (GPx) and catalase (CAT) as well as altered levels of enzyme cofactors such as zinc, copper, selenium and iron have been reported in SCA patients without and with microalbuminuria [5]. The knowledge of the association of microalbuminuria with oxidative stress could be of therapeutic interest. Although the use of antioxidants and supplementation of enzyme cofactors can be conceivably envisaged, the prominent renoprotective effect of angiotensin II type 1 receptor blocker (ARB) Telmisartan in type 2 diabetes, a condition associated with microalbuminuria as SCA has been reported to be mediated through enhancing antioxidant defense capacity and reducing oxidative stress [6]. Unfortunately, data on oxidative stress in SCA patients with and without microalbuminuria are very scare in Africa, a setting where SCA is prevailing [7–10]. In order to contribute to a better understanding of the physiopathology of glomerular damage in SCA, this work aimed to study the relationship between urinary albumin creatinine ratio (UACR) and enzymatic antioxidant status assessed using two markers: serum GPx and Cu-Zn SOD, in Steady state Black African children with SCA.

## Methods

### Subjects and study design

The design of this cross sectional study has been described elsewhere [11]. The study was conducted over a period of three months in two hospitals of Kinshasa/DR Congo: Monkole hospital and Saint-Crispin Medical Center. We consecutively recruited Steady state children aged between 2 and 18 years old with established diagnosis of homozygous SCA by isoelectric focusing method on the Capillaris 2® (SEBIA, France) after written informed consent provided by their legal guardians. Exclusion criteria included a recent history of blood transfusion, a current crisis or history of sickle cell crisis or acute illness two months prior to the study.

### Data collection procedure and laboratory analysis

For each patient, anthropometric parameters, past medical history especially current medication were obtained and physical examination performed.

A venous blood sample was collected for the determination of the level of GPx and Cu-Zn SOD as well as other biochemical parameters of interest including Fetal hemoglobin (HbF), blood count, serum creatinine, total bilirubin and its fractions, lactate dehydrogenase (LDH), iron, ferritin, C reactive protein (CRP), plasma free Hb.

Cu-Zn SOD and GPx were assayed by double-sandwich Elisa method using biotin-streptavidin system [11]. For GPx and Cu-Zn SOD, the blood samples were centrifuged for 10 min at 3000 × g and the obtained plasma was separated, aliquoted to microtubes, and stored frozen at −80 °C until testing a few days later.

Single spot morning urine specimens were collected and urine samples containing blood (1+ or greater), white blood cells (1+ or greater) and/or nitrites were excluded. Both urine dipstick test and UACR measurement were performed. UACR was performed using an immunoassay method with DCA Bayer 2000 reagent (DCA Bayer analyzer®, Siemens Healthcare Diagnostics Pyt Ltd., 885 Mountain Highway, Australia). Normal albuminuria, microalbuminuria and macroalbuminuria were defined as UACR < 30 mg/g, 30 to 299 mg/g and ≥ 300 mg/g, respectively [12].

### Ethical approval

Ethical approval for the study was granted by the institutional review boards of the Monkole hospital (006 CEFA-MONKOLE/2014) in line with the principles of the Declaration of Helsinki, second revision. The aim and study procedures were explained to the parents or legal guardians and they provided written consent before any of the subjects were included.

### Data management and statistical analysis

Statistical analyses were performed using SPSS (version 21.0, SPSS Inc., Chicago, USA). The primary focus in the data analysis was to assess the relationship between UACR and the antioxidant status. For this purpose, bivariate correlation (Pearson’s correlation coefficient) analyses were performed to analyze the relationships between UACR and other numerical data. Analyses were carried out with Pearson’s correlation, simple and multiple linear regressions. Step-wise multivariate regression analysis was performed to determine the independent variables for UACR. For other results, numerical variables are presented as mean ± standard deviation (SD) for normally distributed values or as median with interquartile (IQ) range for nonparametric values and categorical variables are presented as percentage. Pearson chi-square or Fisher’s exact test were used to assess differences in categorical data between groups and Student *t*-test or Mann Whitney *U* test were used to assess numerical data as appropriate. Statistical significance was defined as *p* value of < 0.05.

## Results

### General characteristics of patients

Table 1 shows the general characteristics of the study population. Seventy children with SCA in steady state (56% boys) were selected among which 68 had UACR results. Their mean age was 9.9 ± 4.3 years. Macroalbuminuria was not found in this series. Prevalence of microalbuminuria was 11.8% in the whole group. Microalbuminuria was more prevalent among girls than boys, however, there was no statistically difference between the two groups (17.2% versus 7.7%; *p* = 0.293.

**Table 1:** General characteristics of the study population

	Whole group *n* = 70	Boys *n* = 39	Girls *n* = 31	*p*
Age, years	9.9 ± 4.4	10.5 ± 4.3	9.0 ± 4.4	0.153
Hb, g/dl	8.2 ± 1.3	7.9 ± 1.3	8.6 ± 1.4	0.040
Leukocytes, elts/mm^3^	11618 ± 4621	11339 ± 4114	12011 ± 5113	0.568
Platelets, elts/mm^3^	380727 ± 154522	364.358 ± 132.846	401.884 ± 184.553	0.344
Reticulocytes, %	12.0 (8.5–17.6)	11,0 (7,0–15,5)	14,0 (9,6–19,8)	0.117
HbF, %	8.7 (3.5–16.9)	13.9 (6.9–21.4)	6.2 (2.1–9.7)	0.004
Patients receiving HU, %	37	21	16	0.552
HbF in patients receiving HU, %	14 (7–21)	11 (6–18)	19 (13–25)	0.081
Indirect Bilirubin, mg/dl	1.9 (0.9–3.5)	1.5 (0.9–3.8)	1.9 (1.1–3.5)	0.941
Total Bilirubin, mg/dl	2.5 (1.5–4.0)	2.2 (1.3–4.3)	2.6 (1.7–4.0)	0.632
Creatinine, mg/dl	0.38 ± 1.05	0.38 ± 0.12	0.37 ± 0.11	0.600
GFR, ml/min/1.73 m^2^	211 ± 54	209 ± 67	215 ± 35	0.691
LDH, UI/l	544 (400–771)	457 (338–761)	653 (490–788)	0.012
CPR, mg/l	3.4 (2.0–5.1)	3.0 (1.3–4.5)	3.9 (2.8–6.9)	0.151
Iron, micromol/l	16.2 (13.2–19.2)	16.3 (14.0–21.1)	15.5 (11.5–17.8)	0.892
Ferritin, ng/ml	209 (106–453)	301 (111–613)	184 (104–378)	0.396
Free plasmatic Hb, mg/l	168 (116–267)	140 (120–240)	210 (106–288)	0.278
Cu-Zn SOD, pg/ml	305 (112–556)	281 (96–426)	360 (130–657)	0.803
GPx, micromol/l	220 (100–476)	242 (107–549)	210 (93–466)	0.699
UACR, mg/g	12 (9–22)	10 (9–20)	18 (10–26)	0.058

Values are presented as means ± SD, median (IQ 25–75) or absolute frequency

*CRP* C reactive protein, *HU* hydroxyurea, *GFR* glomerular filtration rate, *Hb* hemoglobin, *HbF* fetal hemoglobin, *LDH* lactate deshydogenase, *GPx* Gluthation peroxidase, *SOD* Superoxide dismutase, *UACR* Urinary albumin creatinine ratio

In the study group, 37 patients (53%) received hydroxyurea (HU). Compared with the untreated patients, those who received HU had higher rates of GPx and Cu-SOD. They also had lower levels of UACR but the difference was not statistically significant (Table 2).

**Table 2:** Comparison of rates of antioxidants and albuminuria based on the HU treatment

	Treated (37)	Untreated (33)	*P*
GPx, micromol/l	391 (199–678)	108 (65–220)	<0.001
Cu-Zn SOD, pg/ml	402 (96–694)	132 (53–407)	0.139
UACR, mg/g	10 (9–17)	19 (10–27)	0.094

Values are presented as median (IQ 25–75)

*GPx* Gluthation peroxidase, *SOD* Superoxide dismutase, *UACR* Urinary albumin creatinine ratio

### Correlation between UACR and study variables

Table 3 shows correlation between UACR and study variables. LDH (*r* = 0.260; *p* = 0.033) and leucocytes levels (*r* = 0.264; *p* = 0.033) were positively correlated with UACR whereas GPx (− 0.328; *p* = 0.007) and Cu-Zn SOD (− 0.210; *p* = 0.091) were negatively correlated with UACR. There was no statistically correlation between UACR and age, systolic blood pressure, diastolic blood pressure, creatinine, glomerular filtration, fetal hemoglobin, plasma free hemoglobin, serum iron, ferritin, indirect bilirubin and CRP.

**Table 3:** Correlation between UACR and other variables in patients studied

Variables	UACR mg/g	
	*r*	*p* value
GPx, micromol/l	−0.328	0.007
Cu-Zn SOD, pg/ml	−0.210	0.091
Age, years	−0.135	0.271
SBP, mmHg	0.224	0.078
DBP, mmHg	0.110	0.389
Leucocytes/mm3	0.264	0.033
Fetal Hb, %	−0.011	0.931
LDH, UI/l	0.260	0.033
Free plasmatic hemoglobin, mg/dl	0.139	0.264
Indirect bilirubin, mg/dl	−0.016	0.898
CRP, mg/l	−0.002	0.988
Serum iron, micromol/l	−0.009	0.940
Ferritin, ng/ml	−0.104	0.397

### Linear regression considering UACR as dependent variable

**Fig. 1:**
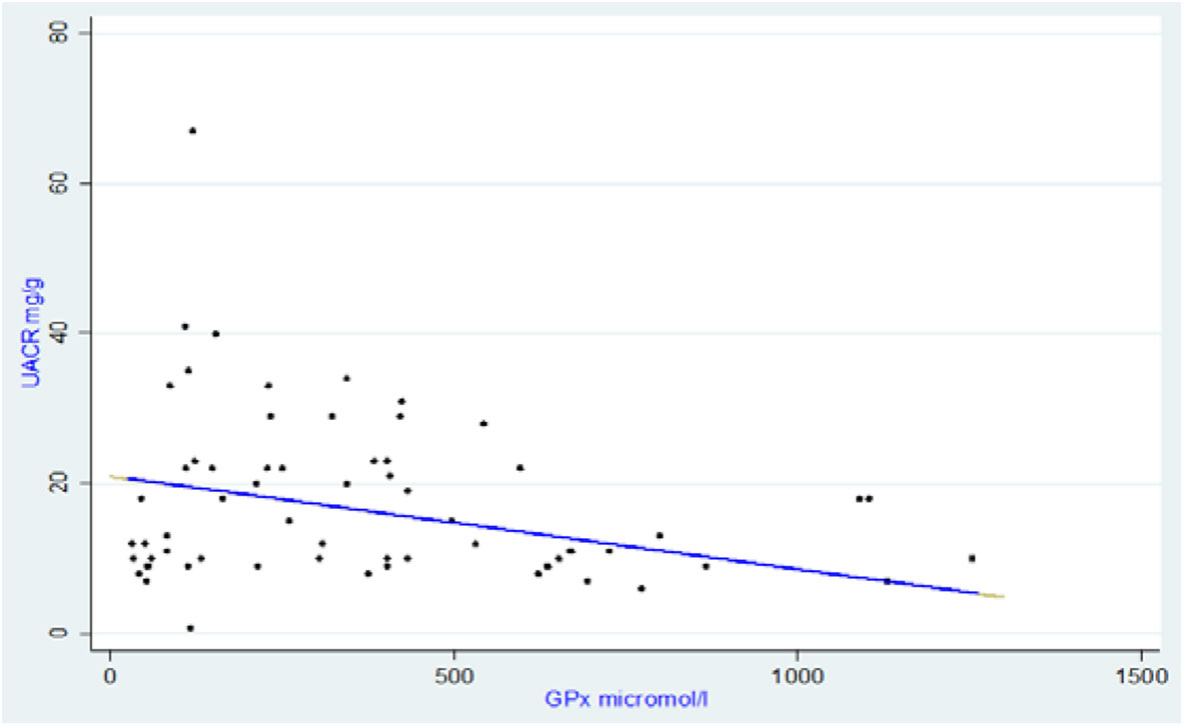
UACR as a function of GPx

**Fig. 2:**
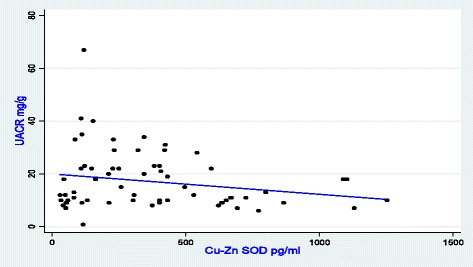
UACR as a function of Cu-Zn SOD

The results related to the linear regression are reported in Table 4 and Figs. 1 and 2. Simple linear regression indicates that GPx, leucocytes levels and LDH respectively explain 10.8, 7.0 and 6.8% of the variation of UACR. The Cu-Zn SOD influences the variation of UACR by about 4.4% without reaching statistical significance. In multivariate analysis, only GPx and LDH were associated with UACR. After adjustment, these two factors account for 14.4% of change in UACR.

**Table 4:** Simple and Multiple linear Regression of UACR according to other variables of interest in patients studied

Variables	constant	ß coefficient	*P* value	*R* ^2^
Simple linear regression				
GPx, micromol/l	20.955	−0.012	0.007	0.108
Cu-Zn SOD, pg/ml	19.943	−0.008	0.091	0.044
Leucocytes/mm^3^	9.802	0.001	0.033	0.070
LDH, UI/l	10.917	0.011	0.033	0.068
Multiple linear regression	14.727			0.144
GPx, micromol/l		−0.010	0.049	
LDH, UI/l		0.010	0.046

## Discussion

The main findings of the present cross-sectional study of Steady state SCA children can be summarized as follows. First, albuminuria was inversely correlated with antioxidant enzyme levels and positively to absolute white blood cell (WBC) count and LDH enzyme levels. Second, antioxidant enzyme glutathione peroxidase (GPx) and LDH levels emerged as the main independent multivariate determinants of albuminuria but the model explained only 14% of the variations of albuminuria. Third, patients receiving hydroxyurea appear to have improved antioxidant capacity and low UACR.

In our cohort, we found an inverse correlation between albuminuria and antioxidant enzyme GPx and Cu-Zn SOD. This observation is similar to previous reports from Nigeria [8], Egypt [9] and Qatar [5]. The decrease in antioxidant enzyme levels and activity could be explained by their susceptibility to ROS-induced oxidation [13, 14]. Reduced glutathione (GSH), a cofactor for GPx, is oxidized to glutathione disulfide (GSSG) through its reduction of free radicals and ROS and is an essential element to reduce hydrogen peroxide (H_2_0_2_). This finding may suggest that the overabundance of oxidative stress leads to consumption or inactivation this protective cofactor [13, 14]. Oxidative damage may alter both the structure and the function of the glomerulus due to its effects on mesangial and endothelial cells [8, 15]. Oxidative stress has been reported to oxidize angiotensinogen and convert it to angiotensin II (ATII) and to increase secondary angiotensin type 1 receptor (AT1R)-mediated generation of transforming growth factor-beta (TGFβ) in the kidney in SCA; excessive AT1R signaling causes SCA glomerulopathy [15, 16].

A Previous study reported the relationship between albuminuria and inflammation, and hemolysis [17]. This report has been confirmed by our results, albuminuria was positively correlated with leukocytes count and LDH level. These results suggest that the activation of vascular endothelial cells and the circulating blood cells represent the continual inflammation seen in SCA. Upon activation, circulating white blood cells and platelets express adhesion glycoproteins leading that will interact with endothelial cell adhesion molecules leading consequently to endothelial dysfunction [14].

The LDH is known to be a marker of hemolysis. The positive association between albuminuria and LDH suggest the central role of hemolysis as the starting point for many of the subsequent complications of SCA, including kidney damage [14, 18]. A Previous study has already reported a significant correlation between serum LDH levels and albuminuria [19]. Indeed, hemolysis through increased plasma-free hemoglobin concentrations can induce the generation of ROS by non-enzymatic (Fenton reaction) with subsequent oxidative stress, inflammation, endothelial dysfunction and tissue damage [14, 19]. However, the hemolytic origin of circulating LDH remains a matter of controversy. Indeed, Neely et al. found that the increase in serum LDL levels was not correlated with plasma-free hemoglobin level and suggested that the source of LDH is damaged tissue as this enzyme is an ubiquitous one and seen in nearly all living cells where it catalyzes the conversion of lactate to pyruvic acid [20].

In multivariate analysis, GPx and LDH levels were independent determinants of albuminuria but the model explained only 14% of the variation in albuminuria levels suggesting the non-inclusion in the model of other variables susceptible to influence this variation. Although an association between markers of oxidative stress and common secondary diseases in SCA have been reported, other factors such as diet, physical activity, and other comorbid diseases associated with SCA may interfere with the variations in albuminuria levels [14]. Some of these factors were not included in the present study and could explain the low determination coefficient of the multivariate model.

For decades, many studies worldwide have shown the benefit of HU in SCA [21, 22]. Although the difference did not reach statistical significance, patients who received HU had higher levels of Cu-Zn SOD and lower UACR. Unlike GPx in both groups (under HU patients and untreated patients) had reached statistical significance. The precise mechanism by which HU produces its varied effects is not fully elucidated. The efficacy of HU is generally attributed to its ability to boost the levels of HbF [23].

Our results should be interpreted within the limitations of the present work. First, the cross-sectional design of the study precludes the establishment of temporal relationships between study variables. Secondly, the small sample does not confer much power to statistics tests to identify additional associations between the study variables. Third, given the limited financial resources, we did not measure the cofactors of antioxidant enzymes and trace elements to better explain the alterations in enzymes levels. The assay of transferrin also was not done. However, we can mention that both ferritin (evaluated in present study) and transferrin prevent the iron to react with its immediate environment. Indeed, in healthy cells, the iron ions are chelated by transport proteins (transferrin) or storage proteins (ferritin). Fourth, in medical human literature, there are few data on reference values of each antioxidant or oxidative stress markers. It is known that, initially, the body will react in a moderate oxidative stress by overexpressing antioxidant enzymes (eg when exercise). If the stress persists and produces massively free radicals, SOD and GPx will be destroyed and its concentration drops. Paradoxically, a too high concentration of SOD can be dangerous because, in this case, it is the basis of a hydrogen peroxide overproduction (paradoxical effect of antioxidants) [24].

## Conclusion

In the present study, albuminuria was associated with decreased antioxidant capacity and increased levels of markers of hemolysis and inflammation. Therefore, strategies targeting the reduction of Sickling and subsequent hemolysis and oxidative stress and inflammation could help preventing or at least delaying the progression of cardiovascular and kidney disease in SCA children.

## Abbreviations

ARB: Angiotensin II type 1 receptor blocker
ATII: Angiotensin II
CAT: Catalase
CRP: C reactive protein
GPx: Glutathione peroxidase
HbF: Fetal hemoglobin
HU: Hydroxyurea
LDH: Lactate dehydrogenase
ROS: Reactive oxygen species
SCA: Sickle cell anemia
SOD: Super oxide dismutase
TGF β: Transforming growth factor beta
UACR: Urinary albumin creatinine ratio
WBC: White blood cell
